# The Pore-Domain of TRPA1 Mediates the Inhibitory Effect of the Antagonist 6-Methyl-5-(2-(trifluoromethyl)phenyl)-1H-indazole

**DOI:** 10.1371/journal.pone.0106776

**Published:** 2014-09-02

**Authors:** Hans Moldenhauer, Ramon Latorre, Jörg Grandl

**Affiliations:** 1 Centro Interdisciplinario de Neurociencias de Valparaíso, Facultad de Ciencias, Universidad de Valparaíso, Valparaiso, Chile; 2 Department of Neurobiology, Duke University Medical Center, Durham, North Carolina, United States of America; Indiana University School of Medicine, United States of America

## Abstract

The transient receptor potential ion channel TRPA1 confers the ability to detect tissue damaging chemicals to sensory neurons and as a result mediates chemical nociception *in vivo*. Mouse TRPA1 is activated by electrophilic compounds such as mustard-oil and several physical stimuli such as cold temperature. Due to its sensory function inhibition of TRPA1 activity might provide an effective treatment against chronic and inflammatory pain. Therefore, TRPA1 has become a target for the development of analgesic drugs. 6-Methyl-5-(2-(trifluoromethyl)phenyl)-1H-indazole (Compound 31) has been identified by a chemical screen and lead optimization as an inhibitor of chemical activation of TRPA1. However, the structures or domains of TRPA1 that mediate the inhibitory effect of Compound 31 are unknown. Here, we screened 12,000 random mutant clones of mouse TRPA1 for their sensitivity to mustard-oil and the ability of Compound 31 to inhibit chemical activation by mustard-oil. In addition, we separately screened this mutant library while stimulating it with cold temperatures. We found that the single-point mutation I624N in the N-terminus of TRPA1 specifically affects the sensitivity to mustard-oil, but not to cold temperatures. This is evidence that sensitivity of TRPA1 to chemicals and cold temperatures is conveyed by separable mechanisms. We also identified five mutations located within the pore domain that cause loss of inhibition by Compound 31. This result demonstrates that the pore-domain is a regulator of chemical activation and suggests that Compound 31 might be acting directly on the pore-domain.

## Introduction

The transient receptor potential ion channel TRPA1 is activated by a wide variety of endogenous and environmental ligands. In addition, TRPA1 is sensitive to transmembrane voltage, ions such as calcium and zinc and temperature [1]–[7]. Physiologically TRPA1 acts as a broad sensor of tissue-damaging stimuli and mice that lack TRPA1 have impaired chemical nociception [8]–[10]. Therefore TRPA1 is a target for the development of analgesic drugs [11], [12]. 6-Methyl-5-(2-(trifluoromethyl)phenyl)-1H-indazole (Compound 31) is an antagonist of mouse TRPA1 that reverses chemically-induced hyperalgesia and allodynia in mice, while leaving core body temperature unaffected [13]. What specific domains or residues of TRPA1 constitute the binding site of Compound 31 is unknown.

Due to the electrophilic nature of many TRPA1 agonists several N-terminal cysteines were readily identified as sites for non-covalent modification by these compounds [8], [14]. In addition, chimeric approaches have been used extensively to identify stimulus-specific domains in TRPA1 [15]–[20]. However, chimeric studies are limited by their requirement for highly similar sequence orthologues that respond differently to a given stimulus. Compound 31 acts as an antagonist on human, mouse and rat TRPA1, making a chimeric approach based on these channels impossible.

To find residues in TRPA1 that mediate activation by temperature, mustard-oil (MO) or the inhibitory effect of Compound 31 we generated a library of ∼12,000 random mutant clones of mouse TRPA1 [21]. We transiently transfected this mutant library into HEK293 cells and loaded the cells with the calcium-sensitive dye fluo-3 to measure channel-activity in a FLIPR-Tetra plate reader. We screened this mutant library while challenging cells with a cold stimulus (25°C−10°C−25°C). In addition, we separately screened this mutant library for activation by 100 µM MO and subsequent inhibition by 1 µM Compound 31. While ∼48% of all clones have a reduced response to both MO and cold temperature, we found one single-point mutation in the N-terminal domain specifically affecting activation by MO but not cold temperature. We also identified several point-mutations that affect inhibition by Compound 31, all of which are located in the pore-domain.

## Results

### A single-point mutation specifically affects activation by MO, but not cold temperature

**Figure 1:**
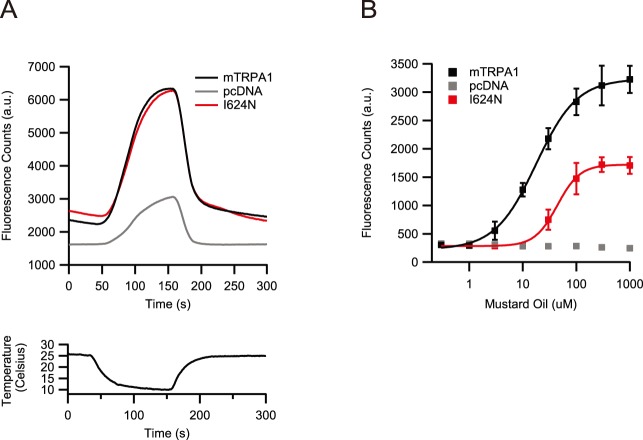
Mutation I624N specifically affects activation by MO. A) Activation profile of HEK293 cells transfected with wild-type mouse TRPA1, pcDNA or mutant I624N upon stimulation with cold temperatures (below). Curves are averages; n = 90. B) Concentration-response curve of wild-type TRPA1, pcDNA or mutant I624N by mustard-oil (final concentrations). Error bars are stdv., n = 6.

We identified one clone from this library that showed a response to cold-temperatures that is identical to that of wild-type TRPA1, but has a substantially decreased response to MO (see Methods for selection criteria). Sequencing revealed two mutations (I624N and F870L) in this clone. We engineered both single-point mutations individually and measured full MO dose-response curves and stimulation by cold temperatures. We found that point-mutation I624N was exclusively responsible for this phenotype, while the other mutation had no functional effect (data not shown). The response of mutant I624N evoked by the cold-stimulus is not significantly different to wild-type mouse TRPA1 (Student’s t-Test, P>0.1, n = 90), arguing strongly that this mutation does not affect channel expression or unitary conductance (Figure 1A).

However, the response to MO is significantly changed (Figure 1B). Specifically, the EC_50_ of mutant I624N in response to MO is significantly shifted to higher concentrations (44±3 µM) compared to wild-type TRPA1 (18±1 µM) (Student’s t-Test, P<0.05, n = 6) and the efficacy of mutant I624N activation by MO is significantly reduced (wild-type TRPA1 = 100±1%; I624N = 53±1%; Student’s t-Test, P<0.01, n = 6). We conclude that mutation I624N specifically affects chemical activation by MO and that this is evidence that activation of TRPA1 by MO is mechanistically separable from activation by cold temperatures. Notably, this point-mutation is separated by only one amino-acid from residue C622, which has been shown previously to bind electrophilic compounds that activate TRPA1 [8], [14].

### Single-point mutations in pore-domain of TRPA1 affect inhibition by Compound 31

**Figure 2:**
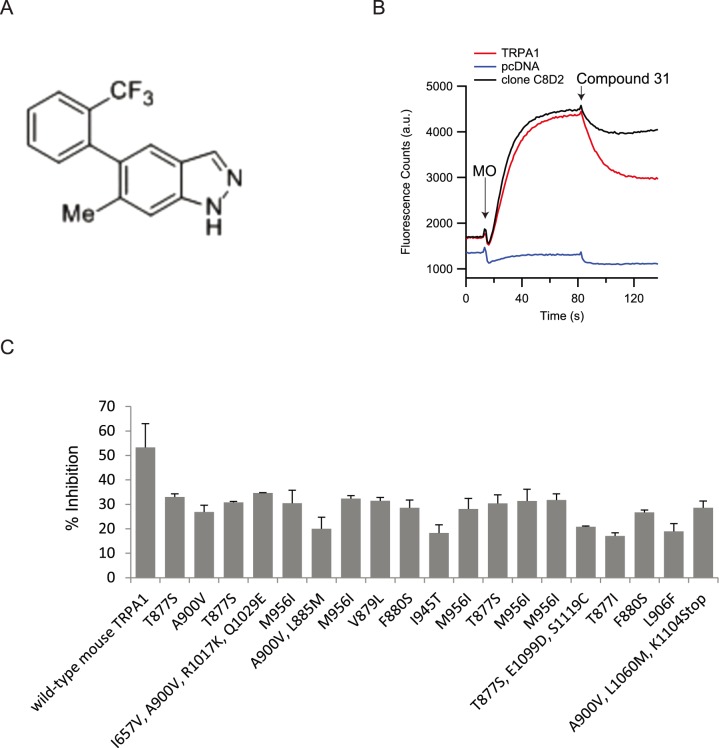
Screen for mutations affecting inhibition by Compound 31. A) Chemical structure of Compound 31 [13]. B) Activation of mouse TRPA1, an example of a mutant clone (clone C8D2; mutation A900V) with reduced inhibition by Compound 31 and pcDNA by 100 µM mustard-oil and subsequent inhibition of 1 µM Compound 31 (final concentrations). Curves are averages, n = 4. C) Average inhibition of mustard-oil (100 µM) induced responses by Compound 31 (1 µM) for 19 clones identified in the initial screen. Single-point mutations identified in each clone are annotated below. Error bars are stdv., n = 3 or 4 for each clone.

**Figure 3:**
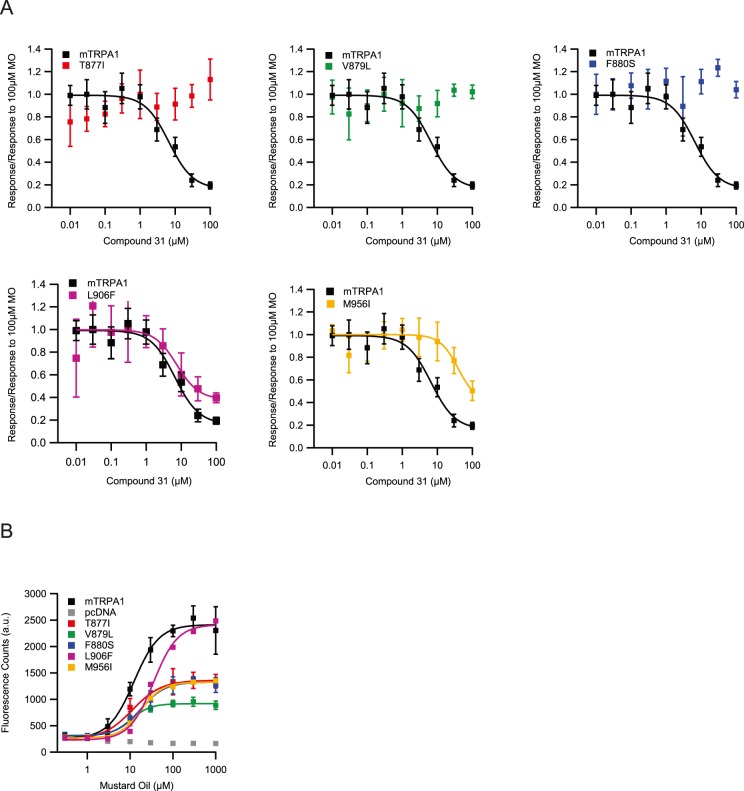
Single-point mutations reduce inhibition by Compound 31. A) Concentration-response curves of wild-type TRPA1 and single-point mutants obtained from pre-incubation of Compound 31 and subsequent stimulation by 100 µM mustard-oil. All curves are normalized to maximal responses evoked by mustard-oil in the absence of Compound 31. Error bars are stdv., n = 6. Lines are fits of Hill-equations to the data. B) Concentration-response curves of wild-type TRPA1, pcDNA and single-point mutants to mustard-oil (final concentrations). Error bars are stdv., n = 4. Lines are fits of Hill-equations to the data.

We also selected, verified and sequenced 19 clones from this library that showed robust sensitivity to MO, but a substantial loss of inhibition by Compound 31 (see Methods for selection criteria) (Figure 2). Notably, all 19 clones contained a point-mutation in one of only seven distinct residues (e.g. residue T877 was mutated to serine in 5 individual clones). For these seven point-mutants we measured full concentration-response curves of inhibition by Compound 31. We found that single-point mutations at five different residues alter the inhibitory effect of 100 µM Compound 31 significantly (Student’s t-Test, P<0.01, n = 6) (Figure 3A). Mutations T877I, V879L and F880S cause a complete loss of inhibition over the concentration-range tested. We conclude that these three amino-acids are essential structures to mediate the inhibitory effect of Compound 31 on TRPA1.

In mutant L906F the IC_50_ was not significantly different (7±6 µM) compared to that of wild-type TRPA1 (6±2 µM) (Student’s t-Test, P>0.5, n = 6), while the uninhibited fraction was significantly increased (38±8%) as compared to wild-type TRPA1 (16±5%) (Student’s t-Test, P<0.01, n = 6). In mutant M956I the IC_50_ was significantly shifted towards higher concentrations (40±79 µM) (Student’s t-Test, P<0.01, n = 6), while a saturating effect of inhibition was not observed within the measured concentration range.

Next, we measured how these identified mutations affect the sensitivity of TRPA1 to MO (Figure 3B). The four mutations T877I, V879L, F880S and M956I are not significantly different in their sensitivity (EC_50_) to MO (wild-type TRPA1 = 22±4 µM; T877I = 11±3 µM; V879L = 10±1 µM; F880S = 18±2 µM; M956I = 18±2 µM) (Student’s t-Test, P>0.1, n = 6), but significantly decrease the maximum efficacy of channel opening (wild-type TRPA1 = 100±4%; T877I = 52±2%; V879L = 34±1%; F880S = 50±2%; M956I = 50±1%) (Student’s t-Test, P<0.01, n = 6). Conversely, mutation L906F causes a significant reduction in sensitivity to MO (wild-type TRPA1 = 22±4 µM; L906F = 35±5 µM) (Student’s t-Test, P<0.01, n = 6), but the maximum efficacy of channel opening is not statistically different (wild-type TRPA1 = 100±4%; L906F = 92±3%) (Student’s t-Test, P>0.01, n = 6).

**Figure 4:**
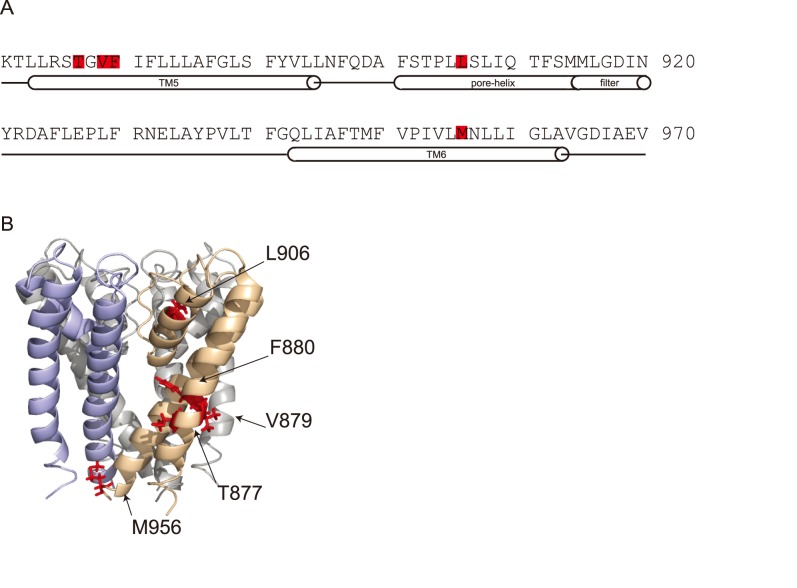
Homology model of the pore-domain of TRPA1. A) Amino-acid sequence of mouse TRPA1 pore-domain. Single-point mutations reducing inhibition by Compound 31 are highlighted in red and structural domains of the pore are indicated below. B) Structural model of the mouse TRPA1 pore-domain. Single-point mutations reducing inhibition by Compound 31 are highlighted in red.

Interestingly, all five amino-acids we identified in our unbiased screen are located within the pore-domain. The three amino-acids that cause complete loss of inhibition (T877, V879 and F880) are adjacent to each other and cluster in the center of TM5. Their residues are predicted to face outside towards the lipid bilayer and not the ion permeation pathway (Figure 4). Residue L906 and M956, which have a partial phenotype, are located in the pore-helix and towards the distal part of TM6, respectively. This pronounced clustering strongly suggests that the pore-domain is the binding-site for Compound 31.

## Discussion

Here, we set out to identify structures in TRPA1 that mediate sensitivity to the electrophilic chemical mustard-oil (MO) and the inhibitor 6-Methyl-5-(2-(trifluoromethyl)phenyl)-1H-indazole (Compound 31). First, we show that a single-point mutation (I624N) in the N-terminal domain specifically affects activation by MO, but not by cold temperatures. The location of this mutation is not surprising, given that it is in proximity to one of the cysteine residues that have been shown previously to be required for MO binding and channel activation [8]. At first this finding is therefore a confirmation of the ability of the screen to identify mechanistically relevant structures. In addition, this is another example showing that in temperature-activated TRP channels the sensitivity to a chemical can be specifically affected without ablating the sensitivity to temperatures [8], [22], [23]. This demonstrates that the mechanism of chemical-activation is functionally separable from the mechanism of temperature-activation. It is however surprising that within our library of 12,000 clones only one was found to have a MO-specific deficit, whereas ∼5600 (48%) of all clones affect channel activation indiscriminately. This result suggests that structures required for MO- and cold-activation are to a large degree, although not completely, identical.

Second, we identified five point-mutations that diminish the inhibitory effect of Compound 31. These five mutations emerged repeatedly from our screening. A likely reason for this robust identification might be that we searched for a loss of function with only a loose requirement for overall receptor function (see Methods for selection details). Another reason might be that the binding-site of Compound 31 is highly intolerant to structural changes. All point-mutants are located in the pore-domain and the mutations causing a complete loss of sensitivity to Compound 31 are focused in the central part of TM5. The most straightforward interpretation of this result is that residues T877, V879 and F880 are an integral part of the binding site of Compound 31. Mutations of residues F906 and M956 might indirectly affect the structure of this binding site and therefore lead to a partial loss of Compound 31 sensitivity. Indeed, a previous study found that residues S876 and T877 determine if high concentrations of menthol are activating or inhibiting TRPA1 [18]. The same homology model we show in Figure 4 had been used in this previous study to demonstrate the possibility that the central pore-domain might form a pocket that could accommodate and perhaps bind menthol [18]. We therefore suggest that Compound 31 is directly acting on this predicted menthol binding-site that is located at the outer side of the pore-domain. It is also likely that a series of aryl-N-(3-(alkylamino)-5-(trifluoromethyl)phenyl)benzamides, that inhibit TRPA1 and that are similar to Compound 31 in their chemical structure, also act on the pore domain on TRPA1 [24]. An alternative interpretation would be that the pore-domain is not directly binding Compound 31, but that it is a structure that regulates channel gating without specificity towards an agonist. The fact that all Compound 31 mutations also affect activation of TRPA1 by MO would be evidence for this.

Regardless, these results demonstrate that unbiased random mutagenesis screens are a powerful tool in identifying sites of chemical interaction. Currently, this knowledge does not contribute directly towards the development of more potent or specific TRPA1 antagonists. However, it is likely that a high-resolution structure of TRPA1 or at least the pore-domain will become available eventually. At this point our data could help designing improved antagonists in a hypothesis driven manner that would not be possible with structural information alone.

## Materials and Methods

### Mutant library generation

The generation of this mutant library was described before [21]. Briefly, a library of 12,000 random mutant clones of mouse TRPA1 was generated by using the GeneMorph II Random Mutagenesis Kit (Strategene). Specifically, from wild-type mouse TRPA1 in a pcDNA3.1(−) we separately mutagenized an N-terminal fragment (∼1,800 bp) and a C-terminal fragment (∼1,500 bp), separated by a unique Cla1 restriction site. For several test reactions 20 randomly selected clones were fully sequenced to adjust PCR conditions to an average mutation rate of 12±2/10,000, which corresponds to ∼2 amino-acid changes per fragment. Subsequently, the respective mutagenized PCR fragments were ligated into wild-typeTRPA1 vector and transformed into XL-10 Gold competent cells. 12,000 clones were selected and Mini-prep DNA prepared as described before [22].

### Cell-culture

HEK293 cells were cultured in HyClone DMEM media (Thermo Scientific) with 10% fetal bovine serum (Life Technologies) and 100 U/ml penicillin-streptomycin (Life Technologies). Cells were harvested by incubation with 0.05 trypsin and resuspension in DMEM. Cells were transiently transfected by adding suspended cells to a mix of DNA, Fugene and Opti-MEM according to manufacturer’s protocol (Promega). For 384-well assays cells were seeded at 400,000/ml (25 µl/well). Cells were incubated at 37°C and 5% CO_2_ for 16–36 hours prior to experiments.

### Library screening

384-well assay plates were washed with HANKS buffer, loaded with fluo-3 and washed with HANKS buffer as described before [25]. The mutant library was screened on a FLIPR Tetra (Molecular Devices) in two independent experiments: First, 100 µM MO followed by 1 µM Compound 31 (final concentrations) was added. Prior to usage DMSO stock solutions of MO and Compound 31 were diluted in HANKS buffer. Second, 384-well assay plates were cooled from 25°C to 10°C using a custom-made device [25].

### Mutant selection and statistical analysis

Average values and standard deviations for MO-activation, Compound 31-inhibition and cold-activation were calculated from positive controls (n = 4 per plate). Plates were analyzed in four groups according to screening days to adjust for day-to-day variation. Mutant clones were selected as MO-specific, if a) mutant MO-response < wild-type – 2x stdv and b) wild-type – 2x stdv < mutant cold-responses. Mutant clones were selected as Compound 31-deficient, if a) mutant MO-response > wild-type – 1x stdv and b) mutant Compound 31-inhibition <35%. Only mutant clones were selected that fulfilled these selection criteria for 3 or 4 wells. For single-point mutants concentration response curves were obtained by exposing cells to various concentrations of Compound 31 and subsequently to 100 µM MO. Concentration-responses were fitted with a Hill-equation:
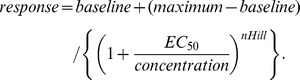



### Structural modeling

The mouse TRPA1 homology model is based on the structure of KcsA (1BL8) and was described before [18].
